# Tensile Mechanical Properties of Swine Cortical Mandibular Bone

**DOI:** 10.1371/journal.pone.0113229

**Published:** 2014-12-02

**Authors:** Tamar Brosh, Doron Rozitsky, Silvia Geron, Raphael Pilo

**Affiliations:** 1 Department of Oral Biology, The Goldschleger School of Dental Medicine, Tel Aviv University, Tel Aviv, Israel; 2 The Goldschleger School of Dental Medicine, Tel Aviv University, Tel Aviv, Israel; 3 Department of Orthodontics, The Goldschleger School of Dental Medicine, Tel Aviv University, Tel Aviv, Israel; 4 Department of Oral Rehabilitation, The Goldschleger School of Dental Medicine, Tel Aviv University, Tel Aviv, Israel; University of Toronto, Canada

## Abstract

Temporary orthodontic mini implants serve as anchorage devices in orthodontic treatments. Often, they are inserted in the jaw bones, between the roots of the teeth. The stability of the mini implants within the bone is one of the major factors affecting their success and, consequently, that of the orthodontic treatment. Bone mechanical properties are important for implant stability. The aim of this study was to determine the tensile properties of the alveolar and basal mandible bones in a swine model. The diametral compression test was employed to study the properties in two orthogonal directions: mesio-distal and occluso-gingival. Small cylindrical cortical bone specimens (2.6 mm diameter, 1.5 mm thickness) were obtained from 7 mandibles using a trephine drill. The sites included different locations (anterior and posterior) and aspects (buccal and lingual) for a total of 16 specimens from each mandible. The load-displacement curves were continuously monitored while loading half of the specimens in the oclluso-gingival direction and half in the mesio-distal direction. The stiffness was calculated from the linear portion of the curve. The mesio-distal direction was 31% stiffer than the occluso-gingival direction. The basal bone was 40% stiffer than the alveolar bone. The posterior zone was 46% stiffer than the anterior zone. The lingual aspect was stiffer than the buccal aspect. Although bone specimens do not behave as brittle materials, the diametral compression test can be adequately used for determining tensile behavior when only small bone specimens can be obtained. In conclusion, to obtain maximal orthodontic mini implant stability, the force components on the implants should be oriented mostly in the mesio-distal direction.

## Introduction

Bones have structure-function relationship. For example, the bones of the lower limb that are loaded extensively during locomotion have superior mechanical properties along their longitudinal axis compared to the lateral axis [1]–[2]. The mandible is a unique bone that differs from long bones in that it has a U-shaped geometry and is connected to the skull bilaterally. It is divided into two parts: the upper bone, which is the alveolar ridge that anchors the teeth, and the lower basal (BAS) bone, the inferior part of which is more condensed and defines the lower border [3].

Mechanically, the jaw bones have to resist natural functional activities, but very often they are also subjected to iatrogenic-induced loadings through dental implants and/or artificial dentures replacing missing teeth. Moreover, in orthodontic treatments, during which non-functional forces are applied to teeth to relocate them, the bones react to these forces by resorption and apposition that ultimately maintain the teeth in their new locations.

One of the side effects of orthodontic treatments is the reaction force system that develops at the anchor teeth and results in undesired teeth movement. To avoid these undesired movements, various alternative implant anchorage techniques were suggested during the early 1990 s, and temporary orthodontic mini implants (OMIs) are currently widely used [4]. In contrast to dental implants that replace natural teeth, which dictate the orientation of the implants in the jaws, OMIs can be inserted in various locations, i.e. in the palate or at the alveolar ridge in the buccal (Buc) or lingual (Lin) aspects, where the long axis of the implant is perpendicular to these planes or at some angle. The loads sustained by these implants are the reaction forces to the applied treatment loads, and their direction is generally perpendicular to the long axis of the implant, primarily resulting in bending forces that act on the OMI heads [5]–[6].

The success rates of OMIs as temporary anchorage devices are high [4], but their success is greatly dependent on the initial stability, which is influenced by the bone quality and quantity (thickness) at the insertion site [7]–[10]. The stresses around a loaded implant and its stability are also influenced by the implant length and diameter [11]–[12]. Moreover, loading direction has been speculated to affect OMI stability [13]–[14]. However, most of the experimental studies attempting to identify the stresses and strains or stability related to OMIs or the surrounding bone have used artificial bone with homogenous density rather than real bones. In addition, in models based on finite elements, the bones have been defined as homogeneous isotropic materials.

Clinical assessments of bone quality and quantity are based on various imaging technologies that provide a general view but lack the capacity to distinguish between mechanical properties in various directions. Bone density [15]–[17] and cortical plate thickness [18] are evaluated by computed tomography (CT) or cone beam CT scans and provide guidelines for OMI placement. Failed OMIs are characterized by lower amounts of total bone volume compared to successful ones [19]. Additionally, it has been noted that more OMI failures occur at locations close to the roots [19]–[20].

An alternative site for OMI placement is often between the roots of the teeth (i.e., interradicular bone). Testing the mechanical properties of the bone at these small sites is challenging. Differences in mechanical properties at various heights of the alveolar bone were reported by Huja et al. 2007 [21] using the micro-indentation technique. However, no effort was made to test the anisotropic nature of the bone at the interradicular site, which is crucial because OMIs can be loaded in various directions, depending on the orthodontic treatment plan. Thus, it can be speculated that different deformations may evolve in the bone due to implant loading in different directions, where high bone deformation may result in OMI loosening and anchorage failure.

Previously, studies have been conducted on mandibles to determine their anisotropic properties. Although the bone specimens were not specifically from the interradicular site, the mechanical properties were superior (i.e., higher elastic modulus) in the mesio-distal (antero-posterior; MD) direction compared to the occluso-gingival (OG) or lateral directions [22]–[23]. It was also shown that the BAS bone is stiffer than the alveolar bone [24]–[25].

The aim of this study was to characterize the anisotropic nature of bone obtained from interradicular sites by measuring tensile stiffness in the MD and OG directions at both the Buc and Lin aspects and from anterior and posterior locations using a swine model and comparing to the BAS bone. The null hypothesis was that the stiffness of the alveolar bone is dependent on both the loading direction and the site from which the specimen is obtained.

## Materials and Methods

In the current study, we used the diametral compression test, also named the Brazilian disk test, which is an experimental method for measuring the tensile strength of brittle materials [26]. In this test, tensile stresses are induced in the plane perpendicular to the applied compressive force. This methodology has previously been used to evaluate properties such as bond strength [27] and to test the influence of dentin tubule orientation on dentin tensile strength [28]. Because bone, like dentin, does not behave as a brittle material, in this study, the dependent variable will be the stiffness during initial loading of the cortical bone of the specimens obtained from the small interradicular sites rather than the tensile strength.

Seven mandibles of 6-month-old (90–100 Kg) domestic swine (Lahav, C.R.O, Israel) were examined. The bones were obtained from a porcine herd. The herd is raised according to the guidelines of the Israeli veterinary authorities (ministry of agriculture) and the Israeli National Care and Use Council (based on the NRC guide), ministry of health. The Ethics Committee of Tel Aviv University specifically approved this study. After slaughter (for meat), the jaws were extracted, carefully debrided from the soft tissues and kept frozen (-20°) wrapped in saline gauzeuntil thawed for specimens’ preparation. The mandibles were cut along the midline using a saw and separated into right and left sides. Cylindrical (r = 1.3 mm) bone specimens were obtained from the cortical bone of each jaw using a trephine drill (trepan bur, model 227A.204.032, Komet, Rock Hill, SC, USA). The thickness was adjusted to 1.5 mm using a polishing disc under X2.5 magnification (Heine, Herrsching, Germany). The final dimensions of each specimen were measured using a digital caliper (Mitutoyo, Tokyo, Japan).

Alveolar interradicular (ALV) bone cylindrical specimens were obtained from two different locations, anterior (Ant) (between 1^st^ and 2^nd^ premolars) and posterior (Post) (between 1^st^ and 2^nd^ molars), both from the Buc and Lin aspects. Identical specimens were obtained from the BAS bone. Eight specimens were obtained from each half of the mandible, and a total of 16 specimens were obtained from each jaw. Before obtaining the specimens, OG lines were drawn using graphite pencil at each site. Based on these lines, the orientation of the specimen related to the OG direction could be controlled while loading in OG or MD directions.

Specimens were kept wet in small containers at 4°C for 2 weeks prior to mechanical testing by the diametral compression test. The specimens were loaded via a universal testing machine (Instron, Model 4502, Buckinghamshire, England) equipped with a 100 N load cell using a crosshead speed of 0.5 mm/min. Force versus deformation data were automatically acquired at 10 Hz. Each specimen was loaded from either the OG or the MD direction (obtained from the right and left sides of each mandible, respectively). The stiffness was calculated from the initial linear portion of the load-deformation curve, obtained from 0–0.05 mm of specimen deformation. As tensile stresses were generated perpendicular to the direction of the applied load, the calculated stiffness was related to the plane perpendicular to the load.

The stiffness values (dependent variable) were statistically analyzed using a 4-way analysis of variance (ANOVA) with repeated measures. The independent variables were stiffness directions (OG vs. MD), bone type (ALV vs. BAS), location (Ant vs. Post) and aspect (Buc vs. Lin). Significant differences were determined based on α = 0.05.

## Results

When OG or MD results are presented, the tensile stiffness is related to these directions.

**Figure 1:**
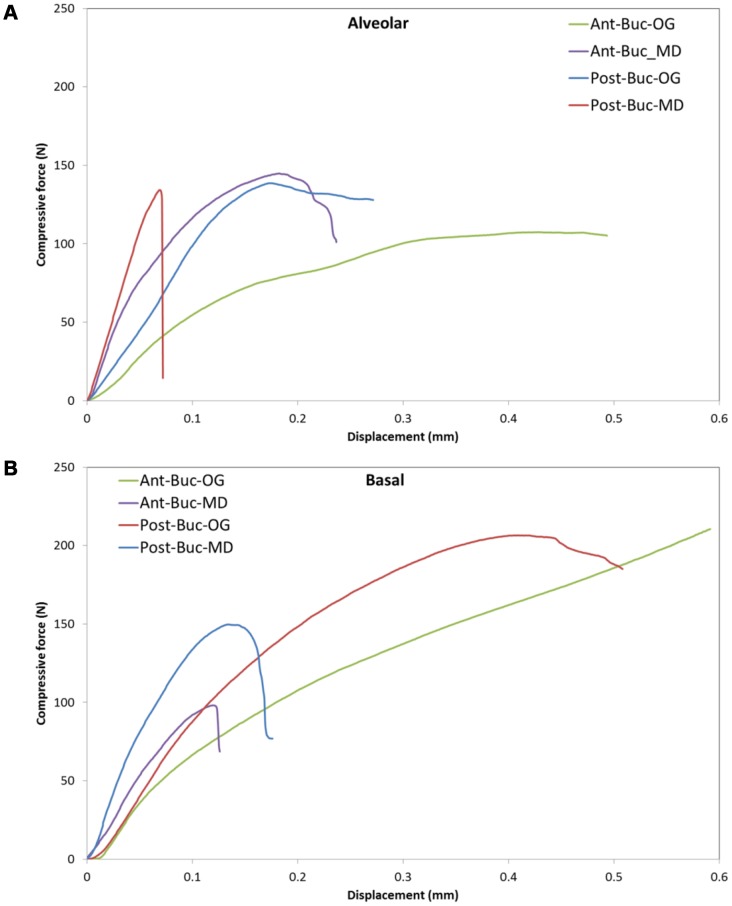
Characteristic load-deformation curves: (a) alveolar, (b) basal. The following abbreviations refer to the specimen location and aspect, and the stiffness related direction (see text): Ant – anterior, Buc – buccal, OG – occluso-gingival, MD – mesio-distal.

Figure 1 (a,b) presents representative load-deformation curves obtained while loading the Buc aspect of the Ant and Post zones in both bone types (ALV and BAS) and directions (OG and MD). In many specimens, a clear peak force could not be detected (e.g., curve Ant-Buc-OG in Fig. 1a). In general, specimens with tension in the OG direction were characterized by predominantly ductile behavior, whereas specimens with tension in the MD direction were characterized by predominantly brittle behavior (Fig. 1).

**Figure 2:**
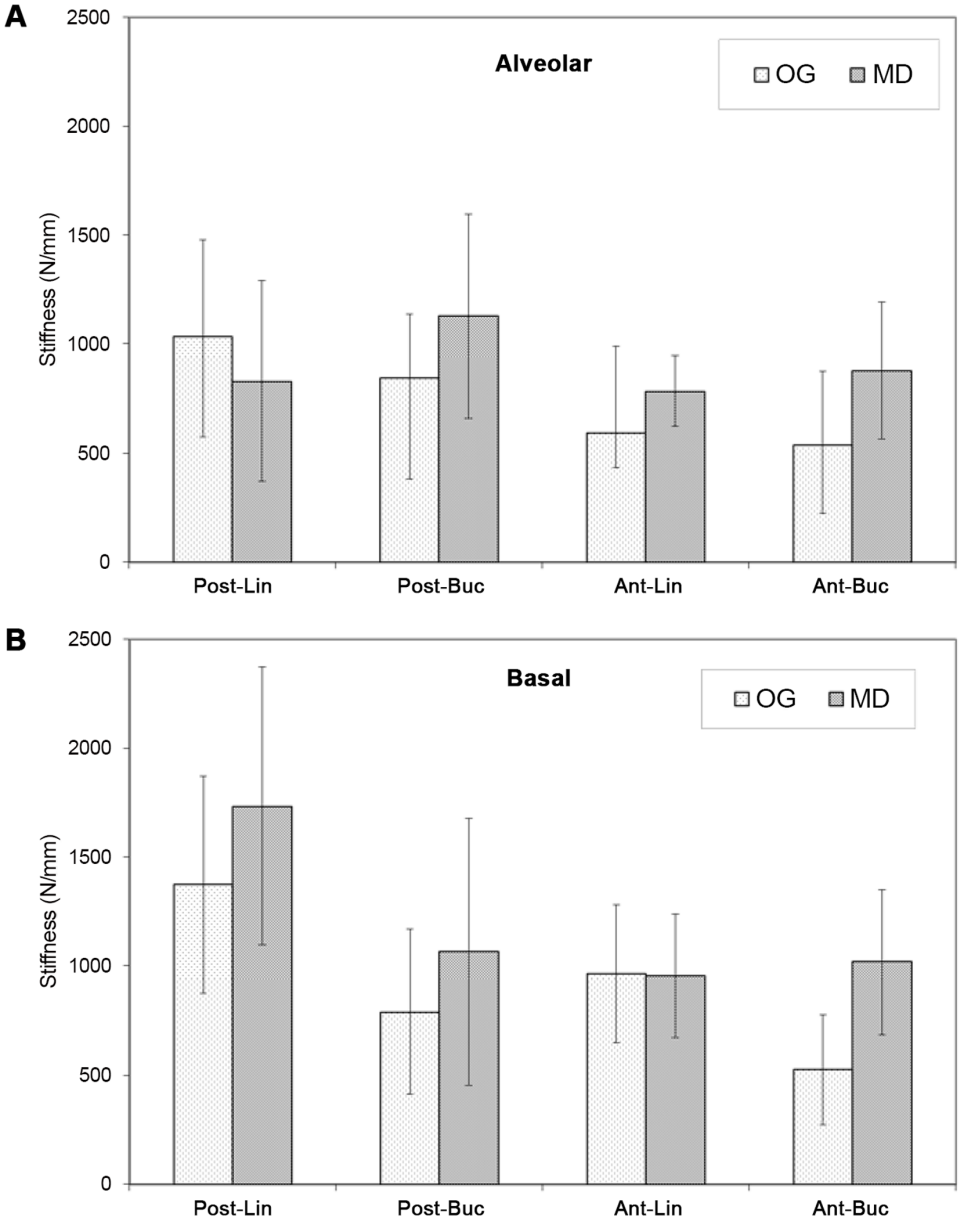
The mean tensile stiffness of (a) alveolar and (b) basal bone. The following abbreviations refer to the specimen location and aspect, and the stiffness-related direction (see text): Ant – anterior, Post – posterior, Buc – buccal, Lin – lingual, OG – occluso-gingival, MD – mesio-distal.

The mean tensile stiffness values (±SD) in the OG and MD directions while loading the ALV (a) and BAS (b) bone are presented in Fig. 2. Values are presented separately for the Ant and Post locations and for the Buc and Lin aspects. The MD values at the BAS and ALV bone ranged between 956 N/mm and 1734 N/mm in the former and between 831 N/mm and 1128 N/mm in the latter. The OG values ranged between 527–1374 N/mm and 538–1035 N/mm in the BAS and ALV bone, respectively. The MD direction was significantly stiffer (31%) than the OG direction (p<0.01). The BAS bone was significantly stiffer (40%) than the ALV bone (p<0.017). The posterior zone was significantly stiffer (46%) than the anterior zone (p<0.001). The Lin zone was stiffer (18.8%) than the Buc zone, although the difference was not statistically significant (p = 0.072).

## Discussion

The first null hypothesis of the current study was accepted, meaning that the tensile stiffness of the interradicular alveolar bone was dependent on both the loading direction and the site from which the specimen was obtained. The diametral compression test was adequate for determining the tensile stiffness of small bone specimens, such as those that can be obtained using bone biopsy tools, from the ALV bone. This site was chosen because it is currently the primary placement area for OMIs. During loading, the circular shape of the specimens was compressed to a more ovular shape, and the load was spread over an area rather than applied at a point. For this reason, ultimate stresses were not calculated, and the tensile stiffness between predetermined deformations was considered. The sensitivity of this test was sufficient to demonstrate the anisotropic nature of this bone, and the MD stiffness was significantly higher than the OG stiffness by 31%. Moreover, the loading curves showed that, when considering the tensile properties, the bone tends to behave as a brittle material in the MD direction and as a ductile material in the OG direction (Fig. 1).

The mandible bone is a unique structure because the functional mastication loads are applied via the teeth. The main component of these loads is parallel to the long axis of the teeth, which is the OG direction. Thus, it could be expected that the bone would have superior mechanical properties in this direction compared to the MD direction. However, because the mandible bone acts as a cantilever that is supported at the temporomandibular joint (TMJ), the functional loads create bending, with maximal stresses in the MD direction. In addition, the larger OG dimension of the mandible compared to the bucco-lingual (lateral) direction is aimed at reducing the bending stresses [29]. Consequently, it is not surprising that the bone is stiffer in the MD direction compared to the OG direction to reduce deformation due to functional forces. This result was valid for both the ALV and the BAS bones. The mechanical properties of bone are related to its hierarchical structure [30]–[31]. The osteons, which are mostly oriented along the curved bone [32], were previously linked to the higher tensile stiffness in this direction compared to other directions, enabling the bone to resist the deflection strains that develop during function.

Our results are in agreement with previous studies that have reported that the mandible bone is stiffer in the MD direction compared to the OG direction. Lettry et al., 2003 [23] used three point bending tests on 20-mm-long specimens. Obviously, such a large specimen size cannot be obtained from the limited ALV bone, making this test inappropriate for evaluating the anisotropy of this zone. Schwartz-Dabney and Dechow (2003) [22] and Chung and Dechow (2011) [2] used an ultrasonic technique on 10-mm-diameter specimens and found that human mandibles have the highest stiffness values mainly parallel to the occlusal plane. Rapoff et al., [2008] [24] performed microindentation tests on one *M. fascicularis* mandible and also found a higher modulus in the antero-posterior direction compared to either the lateral or the OG direction. However, none of the above-mentioned studies considered the specific interradicular small zone bone, which serves as the primary site for OMI insertion.

Significant differences in the mechanical properties of the mandible bone at various sites were detected and confirmed our second hypothesis. The ALV bone was less stiff than the BAS bone, which is in accordance with previous studies [21], [24]–[25]. This behavior represents a biomechanical solution for enhancing the toughness of cortical bone at the alveolar process and close to the roots of the teeth to avoid stress concentrations during occlusal loading [33], [25]. The Post zones and the Lin aspect were stiffer than the Ant and Buc aspects, respectively, as reported in previous studies [34], [22].

Qualitatively, the results obtained in the current study using swine’s mandibles are in accordance to human and other mammal’s mandibles characteristics. Feng and Jasiuk [2010] [35] showed similar results between swine femur cortical bone tested in the laboratory and finite element models of human cortical bone. However, even if the trend of the values of OG as compared to MD is similar between different species, the exact ratios cannot be extrapolated and further studies are necessary. In addition, bone storage prior to testing might influence their mechanical properties. No effect of storage period on mechanical properties after 1 year of storage compared to fresh bones was reported [36]. On the other hand, deterioration in mechanical properties [37] and improvement in strength [38] after freezing were also found. In any case, bones mechanical properties were not tested in vivo and the results are based on ex vivo measurements or evaluation based on imaging data.

Several studies have speculated that OMIs loaded in the OG direction are more prone to failure compared to those that are loaded in the MD direction. In one study, OMIs used for en masse distalization of maxillary teeth had higher success rates than OMIs used for anchorage for molar intrusion [14]. OMIs move during the treatment [39] because the stiffness in the OG direction is smaller compared to that in the MD direction, and the same applied load in that direction will inevitably cause more deformation. Orthodontic forces are applied for a long duration. The viscoelastic behavior of bone, together with the bone remodeling process, is affected by the amount of initial deformation. The outcome of this deformation can lead to loosening and, eventually, OMI failure. The danger of failure is higher at the ALV zone because its stiffness is much lower than the BAS bone, and because this area will deform more for the same amount of load (Fig. 2). Clinically, it has been observed that OMIs placed near the roots have a significantly higher failure rate [19]. It was assumed that less osseous tissue exists at that area, resulting in increased mobility. Histologic data have also emphasized the importance of the bone surrounding the miniscrew [40]. Although it was clinically observed that the root proximity of the miniscrew was a failure factor [20], no relationship with loading direction was considered.

The various locations and anisotropic properties of the mandible bone are not usually applied in biomechanical finite element models testing OMIs [11]. Therefore, it is not surprising that no differences were found by this approach regarding the direction of the force on the von Mises stresses at the cortical bone [41]. The results of the present study emphasize the importance of defining the mandible bone correctly, with different stiffness values for each direction, to obtain reliable outcome strains and stresses in clinically relevant situations [42].

In conclusion, the diametral compression test is an adequate methodology for characterizing the mechanical behavior of small bone specimens. The ALV mandibular bone, similar to the BAS bone, is less stiff in the OG direction compared to the MD direction. Thus, the force components applied on OMIs should be mostly oriented in the MD direction to minimize implant instability.
